# Computational Assessment of Bacterial Protein Structures Indicates a Selection Against Aggregation

**DOI:** 10.3390/cells8080856

**Published:** 2019-08-08

**Authors:** Anita Carija, Francisca Pinheiro, Valentin Iglesias, Salvador Ventura

**Affiliations:** Institut de Biotecnologia i Biomedicina and Departament de Bioquímica i Biologia Molecular, Universitat Autònoma de Barcelona, 08193 Barcelona, Spain

**Keywords:** protein aggregation, protein structure, protein function, cell proteostasis, *Escherichia coli*

## Abstract

The aggregation of proteins compromises cell fitness, either because it titrates functional proteins into non-productive inclusions or because it results in the formation of toxic assemblies. Accordingly, computational proteome-wide analyses suggest that prevention of aggregation upon misfolding plays a key role in sequence evolution. Most proteins spend their lifetimes in a folded state; therefore, it is conceivable that, in addition to sequences, protein structures would have also evolved to minimize the risk of aggregation in their natural environments. By exploiting the AGGRESCAN3D structure-based approach to predict the aggregation propensity of >600 *Escherichia coli* proteins, we show that the structural aggregation propensity of globular proteins is connected with their abundance, length, essentiality, subcellular location and quaternary structure. These data suggest that the avoidance of protein aggregation has contributed to shape the structural properties of proteins in bacterial cells.

## 1. Introduction

Proteins are central components of almost all biological processes, being involved in a variety of complex interactions in the crowded cellular environment [1]. The establishment of non-functional protein–protein interactions has a detrimental impact on cell fitness, both because these contacts sequester proteins into inactive complexes [2] and because it can lead to the aggregation or co-aggregation of proteins into toxic soluble and insoluble assemblies [3]. Importantly, it is increasingly evident that, instead of being an unusual feature of a reduced set of proteins, aggregation is a generic property of many polypeptides [4]. Accordingly, hundreds of unrelated proteins have been reported to aggregate under stress or during ageing [5,6,7]. It has been suggested that this intrinsic propensity to establish anomalous interactions and aggregate is encoded in the amino acid sequence [8,9,10] and, therefore, a variety of complementary methods have been developed to predict those propensities from the linear sequence [11]. Large-scale analysis using these algorithms has led to the hypothesis that proteins have evolved sequence adaptations to counteract their natural propensity to aggregate [12,13,14]. Because in these studies aggregation is analysed along sequences, they mostly measure the aggregation potential of the unfolded state; indeed, the aggregation-prone regions (APRs) that these algorithms identify and evaluate are blocked in properly folded proteins, either because they are buried inside the hydrophobic core or engaged in the series of cooperative non-covalent interactions that sustain the secondary and tertiary protein structure [15]. These sticky sequences might, however, become accessible in case the protein fails to fold due to translational errors. Accordingly, prevention of mistranslation-induced protein misfolding is thought to constraint the evolution of sequences [16,17]. However, it is worth to point out that protein aggregation is not always deleterious and different organisms have exploited the structural/mechanical properties of protein aggregates for functional purposes [18,19].

Protein misfolding is not always a requisite for aggregation, and different proteins have been shown to aggregate from their initial functional conformations [20,21], a propensity that would be exacerbated in the crowded cellular milieu [22]. This suggests that many proteins might be kinetically, but not thermodynamically, stable in their native states under physiological conditions [23,24]. Indeed, the aggregated state of a protein is usually more stable than its native state [25]. According to this view, a solution of proteins that have a certain propensity to aggregate but are correctly folded constitutes a system in a transient state, that might ultimately reach the global free energy minimum of the aggregated state [23]. Therefore, it is conceivable that proteins have evolved strategies to delay/prevent this deleterious transition, adjusting the solubilities of their folded states to those required for function.

In the present work, we address the relationship between the predicted aggregation propensities of protein structures and a set of intrinsic parameters relevant for their function in the cell. To this aim, we exploited a recently developed algorithm, AGGRESCAN3D (A3D) [26,27,28,29], to analyse 619 high-resolution *Escherichia coli* protein structures displaying lower than 40% sequence homology. A3D is an evolution of our previous AGGRESCAN algorithm [30]. The AGGRESCAN amino acid aggregation scale was experimentally derived from protein aggregation measurements in vivo in *E. coli* cells [31]. This placed AGGRESCAN among the most accurate algorithms for the prediction of intracellular protein aggregation, especially when analysing *E. coli* data [32]. A3D also implements these in vivo derived intrinsic propensities but they are strongly modulated by the structural context in which they are located, allowing prediction of the aggregation properties of proteins in their folded states [26,27,28,29]. By analogy to AGGRESCAN, A3D is expected to be accurate when analysing *E. coli* protein structures. Overall, our structural analysis suggests that the avoidance of protein aggregation has contributed to shape the properties of present proteins in this organism.

## 2. Material and Methods

### 2.1. Selection Criteria for the Analysis of Bacterial Proteome

We employed a dataset consisting of 1103 proteins, including, to the best of our knowledge, the most complete set of proteins experimentally detected in the bacterial cytosol at the time we performed the analysis [33]. This is the same dataset we used previously to address the relationship between the aggregation propensity of linear sequences and different protein properties [34], facilitating comparison between both studies. The dataset was used to search through the Protein Data Bank (PDB) [35] in order to identify proteins from the dataset which have available solved crystal/NMR structures. *E. coli* proteins with available three-dimensional structure in PDB format, with X-ray resolution ≤3.5, were considered in this analysis. Furthermore, available three-dimensional structures covering <90% of protein primary sequence were excluded, as well as proteins displaying sequence homology >40%, resulting in a set of 619 bacterial proteins (Table S2). The original 1103 sequences dataset was supposed to correspond only to cytosolic proteins [33], but we could identify also membrane proteins in it (Table S2). Unless otherwise indicated, membrane proteins were not taken into account for the different analysis performed in this work, as they differ significantly from the non-membrane proteins in terms of charge and hydrophobicity.

### 2.2. Structural and Sequential Aggregation Propensity Predictions

Predictions for the structures set were performed using the A3D algorithm which implements structure-based approach and predicts aggregation propensity of initially folded states [26]. Selected proteins were submitted to A3D in the ‘Static mode’ and 10 Ǻ was selected as a distance for aggregation analysis (default sphere radius). The average scores for each protein in the set were obtained. The average score allows comparing the solubility of protein structures differing in size. It also allows assessing changes in solubility promoted by amino acid substitutions in a particular protein structure; this value was assumed to reflect the Structural Aggregation Propensity (STAP) of any given protein. To perform sequential aggregation analysis, we used the linear predictor AGGRESCAN [30] on top of the sequences corresponding to the analysed protein structures. Normalized a4v Sequence Sum for 100 residues (Na4vSS) values were obtained, which reflect the average protein aggregation propensities of the sequences once corrected for their size.

### 2.3. Datasets

Protein abundance data for the set of 619 bacterial proteins was taken from PaxDb [36]. The obtained values were then log_10_ transformed for statistical analysis. Information about protein length, protein subcellular location, number of transmembrane segments and protein active form were obtained from Swiss-Prot Protein knowledgebase [37]. Gene Ontology (GO) enrichment analysis was performed using the Database for Annotation, Visualization and Integrated Discovery (DAVID) [38]. The essentiality of bacterial proteins for cellular fitness was derived from the reported data [39,40] and updated with the data available at server EcoGene 3.0 [41]. The RegulonDB was used to obtain the known *E. coli* operon structure set [42]. To assess the compositional determinants of STAP in the bacterial proteome, amino acids present on the surface of the 10% least and the 10% most aggregation-prone proteins were identified by A3D, and the frequency of appearance of each individual amino acid among the total set of surface amino acids belonging to the two mentioned groups was calculated. Amino acid frequencies for aggregation-prone and soluble *E. coli* proteins were compared using Wilcoxon test.

### 2.4. Definition of the Supersaturation Index (SSI)

The structural supersaturation index (SSI), as the sum:

(1) $$SSI=\frac{\left(C+A\right)}{2}$$

where *C* represents normalized protein concentration and *A* is the normalized STAP score.

The logarithm of the protein abundance levels derived from PaxDb was used in order to determine *C* for each polypeptide from the dataset. These values were then normalized by rescaling them between 0 and 1.

(2) $$C=\frac{\left(Ci-\min\left(Ci\ldots Cn\right)\right)}{\left(\max\left(Ci\ldots Cn\right)-\min\left(Ci\ldots Cn\right)\right)}$$

Cmin = the minimum value of protein concentration from the dataset;

Cmax = the maximum value of protein concentration from the dataset.

The propensity of proteins to aggregate represented by A3D average score was normalized in the same manner by rescaling the values between 0 and 1.

(3) $$A=\frac{\left(Ai-\min\left(Ai\ldots An\right)\right)}{\left(\max\left(Ai\ldots An\right)-\min\left(Ai\ldots An\right)\right)}$$

Amin = the minimum A3D score from the dataset;

Amax = the maximum A3D score from the dataset.

### 2.5. Statistical Analysis

Statistical significance was determined using Wilcoxon test. Analyses were performed using KaleidaGraph software (Synergy Software, Reading, PA, USA). *p*-values < 0.01 were considered statistically significant (* statistically significant at *p* ≤ 0.01; ** statistically significant at *p* ≤ 0.001; *** statistically significant at *p* ≤ 0.0001). The cumulative frequencies plots presented in the manuscript give information on the percentage of proteins (y-axis) that display a value equal or lower to a value, *x*. Fold enrichment indicates how much higher is the proportion of hits in relation to the background sample (*E. coli* proteome). For every GO term, the fold enrichment is the number of positive hits among our list (*n^l^*) between the number of annotated proteins in our list (*p^l^*); and subsequently divided into the rate of hits of that GO term in background (*n^p^*) between the number of total proteins in the background (*p^b^*):

(4) $$Fold\;Enrichment=\frac{\frac{n^{l}}{p^{l}}}{\frac{n^{b}}{p^{b}}}=\frac{n^{l}p^{b}}{n^{b}p^{l}}$$

## 3. Results

### 3.1. A3D Analysis Rationale

**Figure 1:**
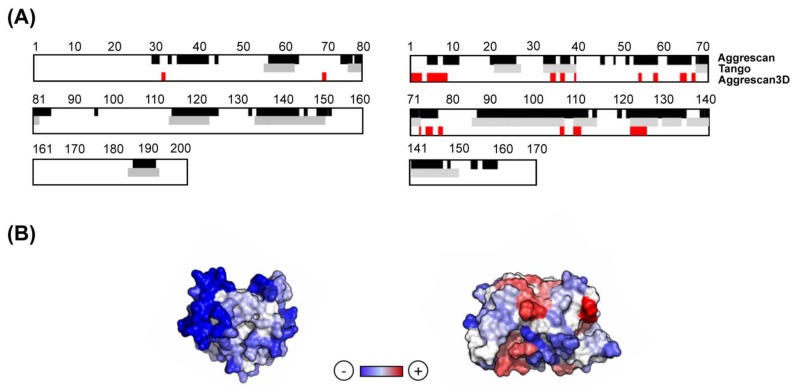
**Analysis of structural aggregation propensity (STAP) for representative soluble and aggregation-prone proteins.** (**A**) Comparison between the aggregation propensity of representative soluble (PDB: 3T36:A; left panel) and aggregation-prone proteins (PDB: 2HO9:A; right panel) using sequence-based predictors and A3D. Aggregation-prone residues are indicated in different colours for each predictor: AGGRESCAN (black), TANGO (grey) and A3D (red). (**B**) A3D analysis of the above mentioned proteins. The A3D average scores for PDBs 3T36:A and 2HO9:A are –1.061 and –0.421, respectively. The protein surface is coloured according to A3D score in a gradient from blue (high-predicted solubility) to white (negligible impact on protein aggregation) to red (high-predicted aggregation propensity).

The A3D algorithm uses the protein 3D structure as an input, which is subsequently energetically minimized using the FoldX force field [43]. Then, an aggregation propensity score is calculated for all the spheres with a 10 Å radius in the protein structure. The variables that contribute to the A3D score are (i) the experimentally-derived individual amino acid aggregation propensities; (ii) the surface area exposure of the amino acids in the sphere; and (iii) the effective distance between adjacent residues and the central amino acid in the sphere. Therefore, the A3D score is structurally corrected and, in contrast to sequence-based aggregation predictors, provides information on the STAP of proteins in their functional folded states [26]. Figure 1 illustrates how A3D allows to discard the contribution of the buried APRs detected by sequence-based algorithms (Figure 1A), and how residues scattered in the sequence can come together in the structure upon folding to form an aggregation-prone surface, as identified by A3D (Figure 1B).

### 3.2. Relationship Between Protein Abundance and Structural Aggregation Propensity

Despite that non-functional interactions might be detrimental, statistically, the large number of non-functional contacts a protein can establish clearly outweighs its functional interactions. This is especially true for abundant proteins, since, according to the law of mass action, the probability to establish a non-functional interaction should be proportional to the protein abundance [2]. Indeed, most protein aggregation processes are strongly dependent on the initial protein concentration [44]. Therefore, an abundant protein with an aggregation-prone surface is expected to be more dangerous than a low-abundant protein with the same surface stickiness [12]. Thus, a negative relationship between protein STAP and protein abundance can be expected. We explored this relationship using *E. coli* as a model organism.

**Figure 2:**
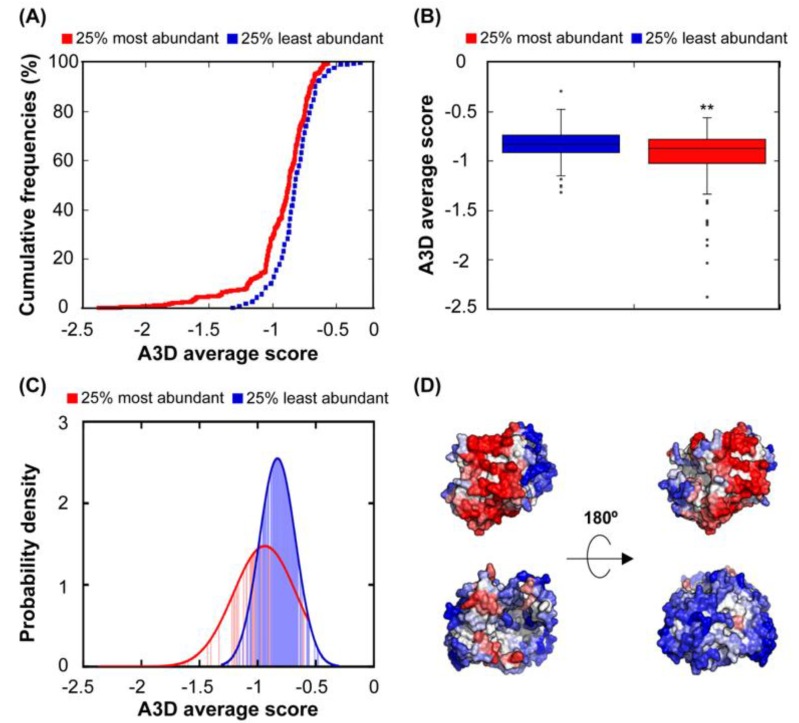
**Relationship between STAP and protein abundance.** (**A**) Cumulative distribution of STAP for the 25% most (red line) and the 25% least (blue line) abundant proteins. (**B**) Box plot showing the comparison between the A3D average score of the 25% most (red) and the 25% least (blue) abundant proteins. (**C**) Probability density function of the 25% most (red) and the 25% least (blue) abundant proteins. (**D**) A3D analysis of representative low-abundance (PDB: 2WSX:A; upper panel) and high-abundance proteins (PDB: 1DFO:A; lower panel). Views of opposite sides of each protein are shown. The A3D average scores for 2WSX:A and 1DFO:A are 0.052 and −0.799, while log_10_ values of their protein abundance are −1.523 and 3.420, respectively. Colour code is as in Figure 1.

We obtained the abundance data for the 612 proteins in our structural dataset and we proceeded by log_10_ transforming the reported abundance for statistical analysis, since the number of mRNAs in the bacterial cytosol encoding a given protein can vary greatly from 1 to 100,000 [45]. The negative correlation between STAP and abundance is low (r = 0.21), but highly significative (*p* = 7.50 × 10^−7^). The comparison of the STAP distribution in the 25% most and least abundant proteins in this dataset is shown in Figure 2A–C and illustrates how highly abundant proteins and proteins present at low concentrations exhibit, indeed, differential aggregation propensities (*p* = 3.26 × 10^−4^), with low abundant proteins displaying surfaces that can support much higher aggregation load than those of high abundant polypeptides (Figure 2D). The probability density function of abundant proteins illustrates the absence of polypeptides with very high aggregation propensity in this group, with a concomitant enrichment in highly soluble proteins (Figure 2C). In addition, low abundant proteins seem not to be under selection for protein aggregation, as the STAP of the 25% of the least abundant proteins is significantly higher than that of the conjunct of the remaining 75% protein structures (*p* = 7.77 × 10^−5^). The higher solubility of abundant proteins would likely work to prevent non-functional interactions in these proteins, even if they become concentrated at specific sub-cytosolic locations.

**Figure 3:**
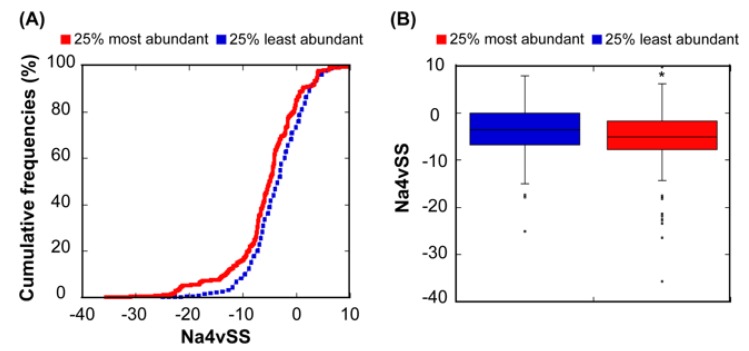
**Relationship between sequential aggregation propensity and protein abundance.** (**A**) Cumulative distribution of Na4vSS values for the 25% most (red line) and the 25% least abundant proteins (dashed blue line). (**B**) Box plot representing the Na4vSS values for the 25% most (red) and the 25% least (blue) abundant proteins.

Different studies have reported a relationship between the abundance of proteins and the aggregation propensity of linear protein sequences [12,34,46,47]. This has been explained in terms of natural selection acting on protein unfolded states in order to minimize aggregation in case of eventual misfolding. In order to test whether this relationship also exists in our dataset, we used our linear aggregation-predictor AGGRESCAN, which uses exactly the same intrinsic amino acid propensities that A3D without taking into account the structural context, to calculate the aggregation properties of the correspondent 612 sequences. As expected, the 25% most abundant proteins display more soluble sequences than the 25% least abundant (Figure 3). However, the significance of the solubility differences between these two subclasses is lower when considering sequences (*p* = 3.91 × 10^−3^) than when we consider structures (*p* = 3.26 × 10^−4^). Thus, it seems that, at least in *E. coli*, in addition to misfolding, structural misassembly, driven by the establishment of non-native interactions between folded protein structures, might also constrain the evolution of proteins [2]. 

Overall, it can be concluded that proteins’ STAP seems to be adjusted to the gene expression levels required for an optimal cell function. Therefore, mutations that decrease the proteins’ surface solubility, or increases in protein expression levels might exacerbate the probability to aggregate [12].

### 3.3. Relationship Between Protein Length and Structural Aggregation Propensity

**Figure 4:**
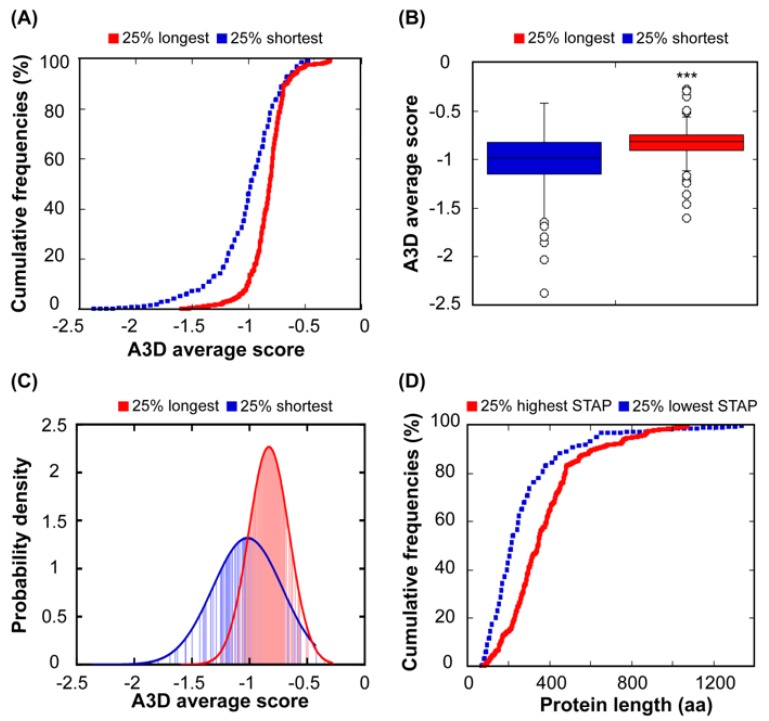
**Relationship between STAP and protein length.** (**A**) Comparison between cumulative distribution of STAP for the 25% longest (red line) and the 25% shortest proteins (blue line). (**B**) Box plot representing the A3D average score of the 25% longest (red) and the 25% shortest (blue) proteins. (**C**) Probability density function of the 25% longest (red) and the 25% shortest proteins (blue). (**D**) Cumulative distribution of protein length for the 25% proteins with the highest STAP (solid line) and the 25% proteins with the lowest STAP (dashed line).

Previous studies have suggested that the length of a given protein might be an important determinant of its aggregation propensity. In this way, it has been shown that longer proteins were more likely to co-aggregate and be sequestered in vivo by artificially designed aggregation-prone polypeptides [48]. Also, it has been reported that the least soluble proteins of three distinct eukaryotic organisms share several common traits, one of them being generally longer in comparison to highly soluble proteins [49]. The comparison of the 25% shortest and the 25% longest polypeptides in our protein set, indicates that both groups exhibit different STAP (*p* = 2.65 × 10^−9^) (Figure 4A–C). Short proteins exhibit a wider distribution of A3D average scores (SD = 0.30), indicating that the dynamic range of aggregation propensities in this group is larger when compared to the group composed of the 25% longest polypeptides (SD = 0.18) (Figure 4C).

To confirm the relationship between size and solubility, we compared the size of the 25% most soluble proteins, according to their A3D average score, and the 25% least soluble. These two subsets correspond to proteins with different size properties (*p* = 9.11 × 10^−7^) (Figure 4D). As expected, the group of low aggregation-prone proteins is enriched in small polypeptides, whereas the proteins in the high aggregation-prone set are clearly larger (Figure 4D). A visual inspection of the aggregation surfaces of short and long proteins suggests that the higher number of exposed aggregation-prone structural patches in the longest proteins could be an important determinant of their higher STAP. The presence of these patches likely responds to the fact that these proteins can interact simultaneously with a larger number of partners or through larger protein interfaces, but this functionality also involves a higher risk of aggregation.

**Figure 5:**
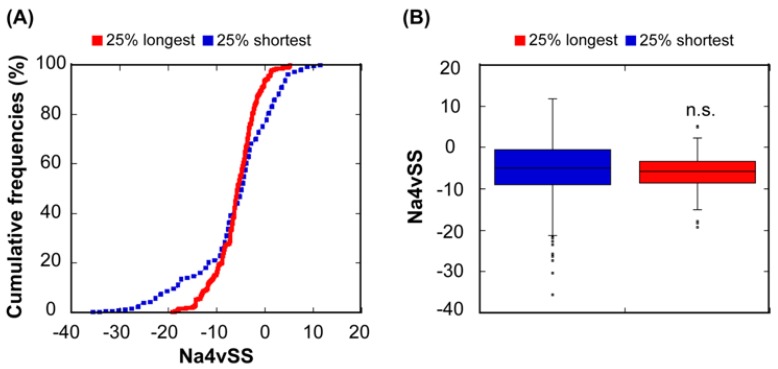
**Relationship between sequential aggregation propensity and protein length.** (**A**) Cumulative distribution of Na4vSS values for the 25% longest (red line) and the 25% shortest proteins (dashed blue line). (**B**) Box plot showing the comparison between the Na4vSS values of the 25% longest (red) and the 25% shortest (blue) proteins.

We used AGGRESCAN to calculate the aggregation propensities of all the protein sequences and then grouped them as described above. The overall aggregation propensities of the 25% shortest and the longest protein sequences do not differ significantly (*p* = 0.72) (Figure 5). The differences observed when considering structural or sequential aggregation propensities are not surprising if we think that many of the sequential aggregation-prone regions are hidden inside the structure when the protein is folded (Figure 1A) and that many structural aggregation-prone regions at the protein surface are composed of residues that are not consecutive in the sequence. In any case, at least in our bacterial protein dataset, the apparent relationship between aggregation and size is best explained if we consider that the selective pressure acts on protein structures instead of/in addition to sequences.

### 3.4. Relationship Between Structural Aggregation Propensity and Protein Function

**Figure 6:**
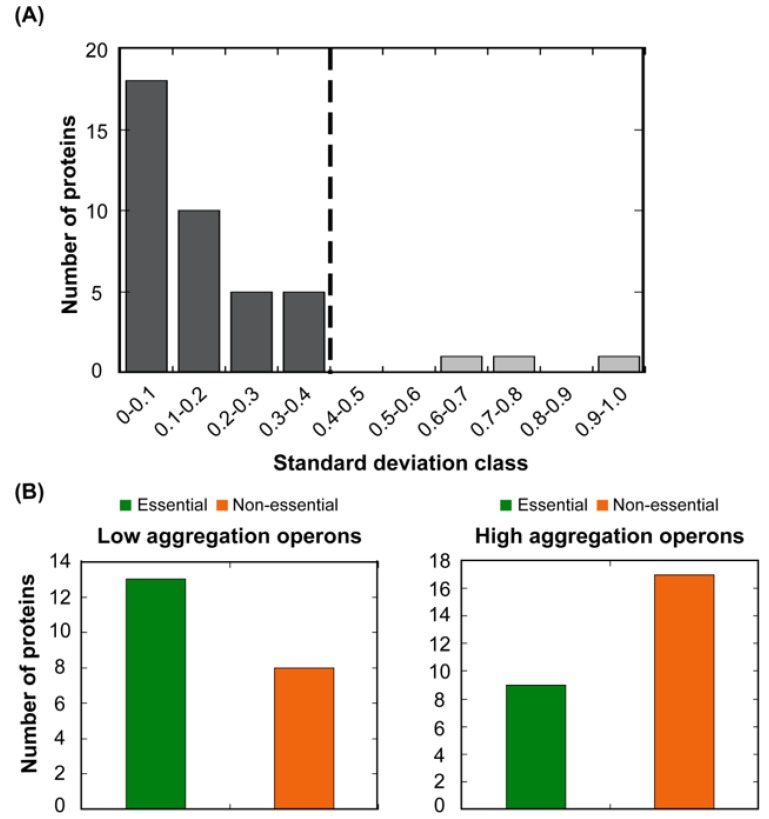
**The variation in A3D average scores for known bacterial operons.** (**A**) The standard deviation of A3D average scores in 41 analysed operons was calculated and operons were divided into 10 classes. The dashed line indicates the standard deviation in the complete set of proteins (0.4). Low standard deviation within an operon indicates that the aggregation propensity of its proteins is similar. (**B**) Number of essential (green) and non-essential (orange) proteins codified by operons with low (left panel) and high (right panel) aggregation propensity.

The set of genes in an operon share a common gene expression regulation and are generally connected by their biological function. As a result, proteins encoded by the same operon are suggested to be present in similar amounts in the cell [33]. The observed association between STAP and abundance would imply that polypeptides in the same operon should have related aggregation properties. To test this hypothesis, we first ascribed the proteins in our complete dataset to individual operons. 234 out of 619 proteins could be ascribed to a particular *E. coli* operon (Table S1). We calculated A3D average scores and, as hypothesized, found out that the standard deviation of the A3D average score between proteins regulated by the same operon is lower in 93% of the cases (38 out of 41) than the standard deviation in the complete set of proteins that could be ascribed to a particular operon (Figure 6).

In the loss of function scenario, the impact of protein aggregation on cellular fitness would be ultimately associated with the particular protein activity. Therefore, it is expected that evolution would select for an overall decreased aggregation propensity in the proteins from operons involved in essential cellular functions [50]. To explore this possibility, the bacterial operons were divided into two groups according to their A3D average scores, those with lower (LA operons) and higher aggregation propensity (HA operons) than the mean propensity of the complete operon protein set (A3D average score = −0.880). The essentiality of approximately half of the proteins in each subset has been annotated via genetic footprinting or knockout experiments [39,40]. Importantly, considering only the annotated polypeptides, the majority of proteins regulated by LA operons are essential, whereas most of those in HA operons are non-essential (Table S1, Figure 6B). This supports the view that, as a trend, the structures of essential bacterial proteins suffer a stronger selection against aggregation than those of non-essential ones.

### 3.5. Effect of Subcellular Location on the Structural Aggregation Propensity

**Figure 7:**
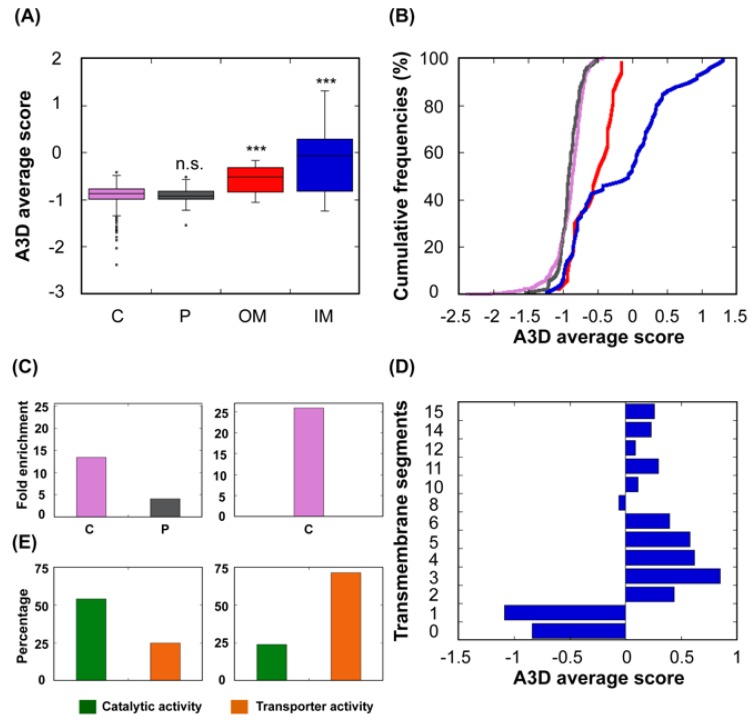
**Relationship between STAP and protein subcellular location.** (**A**) The bacterial proteins were divided into four groups based on the protein localization according to UniProt. (**B**) A cumulative distribution of STAP of proteins located in the cytoplasm (C; purple), periplasm (P; dark grey), outer membrane (OM; red) and inner membrane (IM; blue). (**C**) Proteins with 20% lowest (left panel) and 20% proteins highest STAP (right panel) belonging to the dataset in which proteins with transmembrane segments (TS) were excluded, according to GO terms. Only GO cellular component terms with *p*-value < 0.05 and false discovery rate (FDR) <0.05 were plotted. (**D**) Diagram for the analysed IM proteins showing the number of TS and STAP. (**E**) Analysis of the molecular functions associated to IM proteins with A3D average score <0 (left panel) and A3D average score ≥0 (right panel) according to GO terms proposed by PANTHER.

It is of great importance that the protein maintains its biological function in its native state no matter in which subcellular location this protein resides [46,47,51]. Bacterial proteins can populate other subcellular compartments apart from the cytosol, like the periplasm and the inner and outer membranes. Presumably, their aggregation properties would be adapted to the specific environment in those subcellular locations. A3D analysis shows that proteins residing in the bacterial cytoplasm and the periplasm possess the lowest STAP of the complete dataset (Figure 7A,B). After excluding proteins with transmembrane domains, the distribution of the 20% proteins with the lowest A3D average scores and 20% proteins with the highest A3D average scores in these two compartments was analysed. We found out that soluble proteins were enriched in both the cytosol and the periplasm, whereas enrichment in this last compartment was not observed among the aggregation-prone proteins (Figure 7C). This is consistent with the fact that the periplasm, in contrast to the cytosol, lacks a sophisticated cellular system able to control protein quality and avoid aggregation [52], and is separated from the outside solution by a highly permeable outer membrane that provides limited protection against environmental variations.

**Figure 8:**
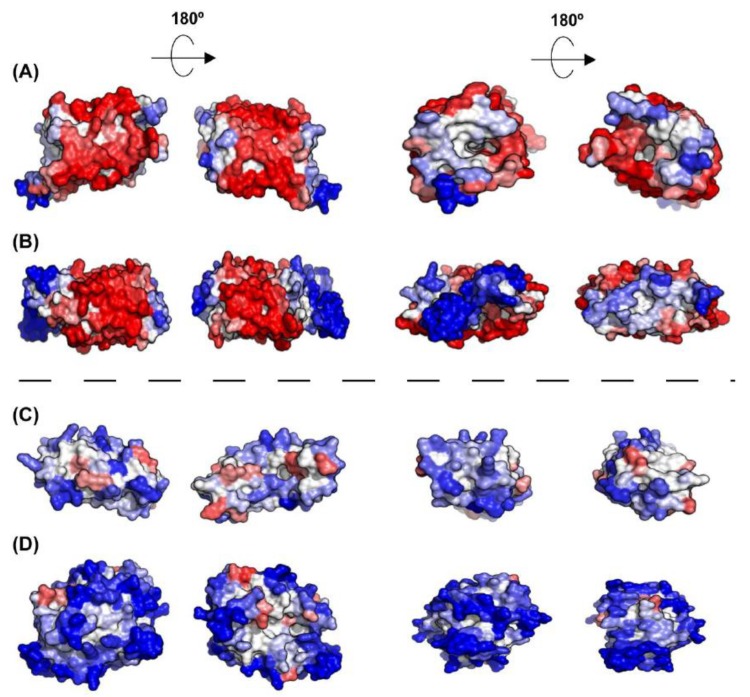
**A3D analysis of selected representative inner membrane proteins.** (**A**) Aquaporin Z (PDB: 1RC2:A; A3D average score = 0.417); (**B**) Nitrate/nitrite transporter NarK (PDB: 4JR9:A; A3D average score = 0.051); (**C**) Acyl-CoA thioester hydrolase YbgC (PDB: 1S5U:A; A3D average score = −0.664); and (**D**) Septum site-determining protein MinD (PDB: 3Q9L:A; A3D average score = −1.031). The dashed line indicates the existence of two protein groups among inner membrane proteins, exhibiting different STAPs. Lateral view and top view of each protein together with the views of opposite sides are shown. Colour code is as in Figure 1.

Not surprisingly, inner membrane (IM) proteins exhibit the highest theoretical aggregation propensities of all bacterial proteins, according to A3D analysis (Figure 7A,B). The gram-negative bacterial inner membrane is a semipermeable shield that preserves the cytoplasm environment. The proteins associated with this membrane can have a variable number of TS per protein [53]. These regions are stable in the hydrophobic environment of this lipid bilayer due to their enrichment in apolar residues. A visual inspection of their structures shows that the highly aggregation-prone surfaces identified by A3D in many of these proteins sharply coincide with regions embedded in the membrane, since the membrane width could be traced in their A3D coloured structure (Figure 8A,B). Interestingly, when the A3D average scores of inner membrane proteins were plotted as a distribution, the existence of two protein groups became evident (Figure 7B). We found that the main difference between these two groups is the number of TS (Figure 7D). In fact, the inner membrane group contains a number of proteins without TS and a low STAP. An analysis of their structures indicates that they are devoid of large aggregation-prone patches suggesting that they associate to the membrane transiently (Figure 8C,D). Proteins with a single TS also display significant predicted solubility, likely because apart from the short TS anchored to the membrane, usually an α-helix, they also exhibit globular domains facing either the cytosol or the periplasm (Figure 7D). Proteins exhibiting two or more TS account for the most aggregation prone structures in the analysed sub-proteome. However, it is worth to mention that these high theoretical propensities do not necessarily translate into a high aggregation in the cell, where IM proteins’ hydrophobic stretches remain protected inside the lipophilic membrane environment.

An analysis of the molecular functions associated to IM proteins with lower and higher predicted aggregation propensity indicates that they seem to play different cellular roles, the low aggregation-prone ones displaying preferentially catalytic activity, and the high aggregation-prone proteins possessing mainly the role of transporters (Figure 7E). 

**Figure 9:**
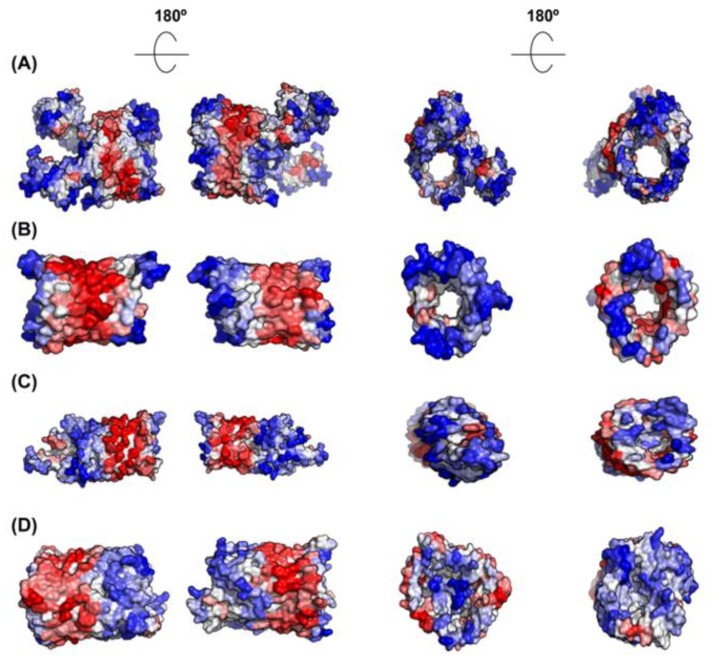
**A3D analysis of selected representative outer membrane proteins.** (**A**) Usher protein FimD (PDB: 3RFZ:B; A3D average score = −0.565); (**B**) Outer membrane protein G (PDB: 2F1C:X; A3D average score = −0.275); (**C**) Long-chain fatty acid transport protein (PDB: 1T16:A; A3D average score = −0.350) and (**D**) Fe(3+) dicitrate transport protein FecA (PDB: 1KMO:A; A3D average score = −0.296). Lateral view and top view of each protein together with the views of opposite sides are shown. Colour code is as in Figure 1.

As IM proteins, outer membrane proteins (OM) are located in a hydrophobic environment, and consequently, they are usually thought to have a high aggregation tendency. However, their STAP lies between those of cytoplasmic and IM proteins (Figure 7A,B). In fact, the outer membrane acts as a permeable barrier to hydrophilic substances. In general, outer membrane proteins display a β-barrel structure that encloses a hydrophilic cavity surrounded by a hydrophobic outer layer embedded in the membrane. A3D structural predictions are able to capture this particular architecture. In the predictions it can be easily seen a high aggregation-prone fringe in the outside, restrained to the exact boundary embedded in the membrane, flanked by low aggregation-prone regions that protrude out of the membrane (Figure 9). Seen from above, or below, it can be observed that the residues flanking the cavity at both inner and outer sides conform surfaces of low aggregation propensity. This particular assembly is achieved by alternating hydrophobic and hydrophilic segments in the sequence [54]. This explains why when linear predictors were used to analyse the aggregation propensities of these proteins, they were wrongly predicted as being even more soluble than the cytoplasmic proteins [34]. In reality, in these proteins, the scattered aggregation-prone regions in the sequence come together in the structure to allow their stable insertion in the membrane, where they remain protected from aggregation.

### 3.6. Compositional Determinants of Protein Structural Aggregation Propensity

**Figure 10:**
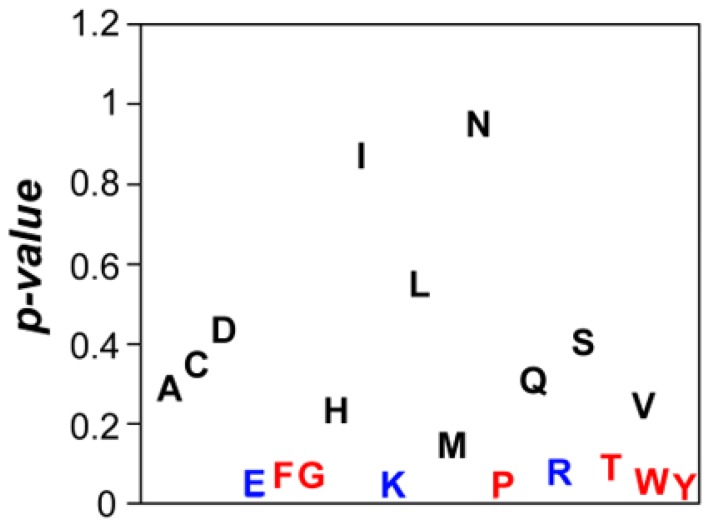
**Comparison of amino acid composition in soluble and aggregation-prone protein surfaces.** Plot of *p*-values correlating surface amino composition for two protein groups: 10% least and 10% most aggregation-prone proteins. In black: amino acids that do not show significant differences. In blue: amino acids that are more abundant in soluble proteins. In red: amino acids that are more abundant in aggregation-prone proteins.

To understand whether there is any compositional bias in the surfaces of the 10% most and 10% least aggregation-prone *E. coli* structures, we compared the frequency of the 20 natural amino acids at their surfaces. Eleven out of twenty amino acids did not show any significant enrichment in any of these two groups (Figure 10). However, we found out that charged residues (glutamic acid, lysine and arginine) were significantly enriched in the soluble structures’ subset (Figure 10). It is generally accepted that charge–charge interactions are important for protein solubility by inducing long-range repulsion between alike-charged species. This has been clearly demonstrated by introducing multiple charged side chains in proteins, a strategy that provides an increased resilience towards aggregation, particularly at elevated temperatures [55,56]. Conversely, neutralization of surface charges is suggested to be required for efficient amyloid fibril formation [57] and chemical and mutational neutralization of charges has indeed been shown to promote aggregation [58]. Importantly, the enrichment in these charged residues is not associated with differences in the pI of soluble and aggregation-prone proteins (*p* = 0.228).

The aromatic residues (phenylalanine, tyrosine and tryptophan) were enriched in aggregation-prone structures (Figure 10). This can be related to the fact that exposed aromatic residues facilitate protein–protein interactions and indeed these three residues are more frequent at protein interfaces than at their surfaces [59]. This property can be partially explained by their ability to establish both π–π or π–cation interactions, their flat surfaces and the entropic benefit of hiding them from water inside interfaces. However, these properties also imply an increased probability of establishing competing non-functional interactions. The trade-off between proper and anomalous interactions will explain why we did not find any hydrophobic aliphatic residue enriched in aggregation-prone structures, since they will increase the aggregation potential without providing a significant counteracting functional advantage. We also identified glycine and proline enriched in the surface of aggregation-prone proteins (Figure 10). These two residues are bad β-sheet formers and their presence might counteract the enrichment in aromatic residues by diminishing the protein probability to form intermolecular β-sheets contacts leading to the formation of protein aggregates [60].

### 3.7. Relationship Between Functional Protein Assemblies and Protein Structural Aggregation Propensity

**Figure 11:**
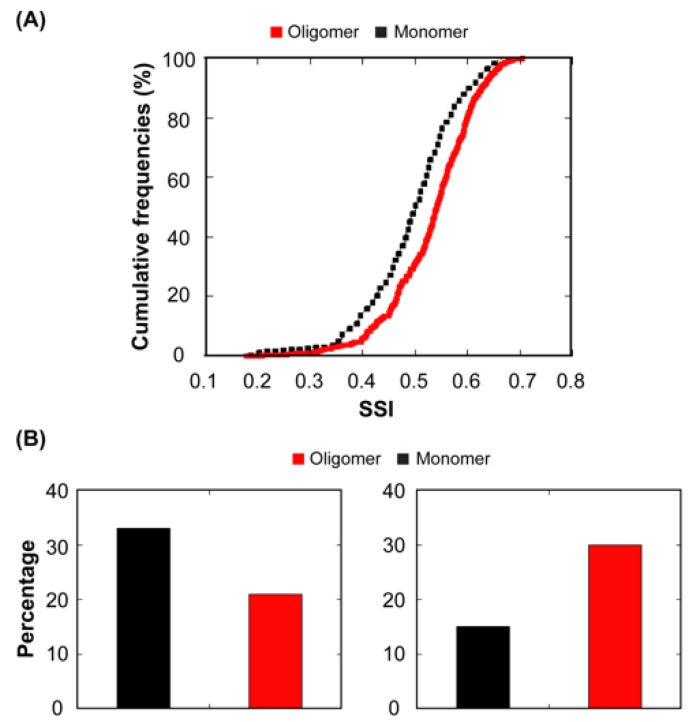
**Relationship between structural supersaturation index and protein active form.** (**A**) A comparison between cumulative distributions of SSI values for the subunits of proteins active as monomers (dashed black line) or as oligomers (solid red line). (**B**) A bar graph representing the percentage of monomers (black) or oligomers (red) from the dataset that display low (left panel) and high (right panel) structural supersaturation indexes (SSI).

As we described above, there is a negative correlation between protein abundance and STAP. Indeed, it has been shown that “supersaturated” proteins, which are maintained at a high concentration relative to their solubility are among the most aggregation susceptible proteins in different proteomes [61]. The presence of supersaturated proteins in the cell may respond to the requirements to exert their biological functions. The stoichiometry of multimeric proteins might imply that their monomeric subunits would be supersaturated relative to proteins that are active in the cell as monomers. We calculated a new parameter, the structural supersaturation index (SSI), for the subunits of oligomeric and for monomeric proteins in our dataset (Figure 11A). This parameter reflects the risk of a folded protein subunit to aggregate at its physiological concentration in the cell. The structures of monomeric proteins displayed significantly lower SSI values than that of oligomeric protein subunits (*p* = 3.29 × 10^−7^). Next, we analysed the oligomeric state of protein subunits displaying the 25% lowest and highest SSI (Figure 11B). The low SSI group contained 33.0% of the monomers and 21.0% of the oligomers in the dataset, respectively. In contrast, the high SSI group encompassed 14.6% of the monomers and 30.0% of the oligomers in the dataset, respectively. Thus, it becomes clear that the formation of multimeric proteins comes at a higher risk of aggregation. Then, why these proteins do not aggregate in vivo under physiological conditions? Mainly, because the sticky interfaces needed for the assembly of the monomeric subunits into a quaternary structure are protected in the native state and accordingly the aggregation risk is only transitory. This implies, however, that cellular conditions or genetic changes that favour multimers dissociation would also favour non-functional interactions, as it occurs in the case of human transthyretin or superoxide dismutase 1, whose dissociation leads to their aggregation and the onset of amyloidosis [62,63].

### 3.8. Protein Structural Aggregation Propensity and Bacterial Symmetric Complexes Self-Assembly

**Figure 12:**
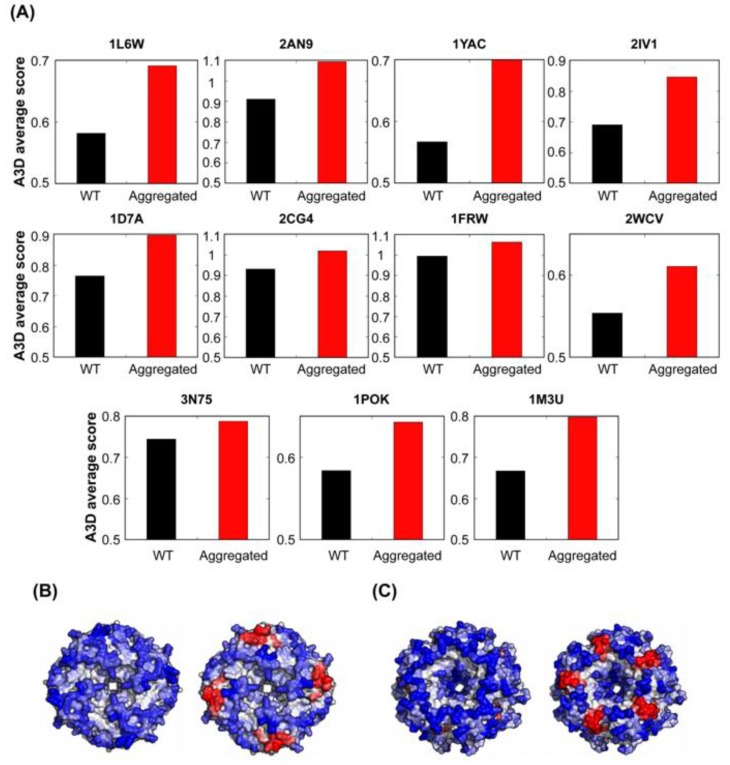
**STAP predictions for bacterial self-assembled homomers with dihedral symmetry and their point mutants that aggregate.** (**A**) Bar graphs for indicated supramolecular complexes comparing A3D average scores for wt proteins and their mutants forming either fibres or focis. Corresponding PDB accession numbers for analysed proteins are indicated on top of the bar graphs. A3D analysis of representative supramolecular assemblies: (**B**) dihedral octamer (PDB: 1YAC; A3D average score = −0.567) and its aggregating mutant (D92L, E94L, K98L, K101L; A3D average score = −0.409) and (**C**) dihedral decamer (PDB: 2IV1; A3D average score = −0.690) and its foci-forming mutant (K24L, K25L, D26L; A3D average score = −0.535). Top-bottom views of each protein are shown. Colour code is as in Figure 1.

A recent study has shown that, even when they are in the native and properly folded states, bacterial symmetric protein complexes are at risk of aggregation [64]. The authors introduced point mutations at the surface of 12 distinct symmetric complexes from *E. coli*, resulting in 73 different variants. Some of these mutants resulted in the formation of aggregates when they were expressed intracellularly. Biophysical measurements and electron microscopy revealed that the aggregated mutants self-assembled in their folded states. Because these mutations were introduced at the surface of the symmetric protein complexes they could indeed impact their STAP. Therefore, we analysed whether A3D predictions were able to detect any difference between the wild type (wt) proteins and those mutants that formed self-assembled entities intracellularly. As it is shown in Figure 12, in all cases, the average STAP of protein complexes forming aggregates was higher than the STAP of the correspondent wt forms. This suggests that the STAP of protein complexes is an important determinant of their assembly and accordingly that mutations impacting this structural property might trigger folded functional complexes to self-assemble into higher-order structures in the cell.

## 4. Discussion

It is now well established that, in addition to stability and function, the avoidance of aggregation is an important driver of the evolution of proteins. However, the large majority of studies that provide support to this hypothesis have measured aggregation along protein sequences [34,46,47]. Therefore, they essentially evaluate the aggregation potential of unfolded or at least partially unfolded states. These conformations are of course relevant for aggregation during or immediately after translation or in the case of eventual misfolding; nevertheless, the time that globular proteins expend in these transient states is relatively short in comparison to the one in which they remain in the native state. 

For a long time, it has been assumed that the attainment of a stable folded conformation in which aggregation-prone sequences are shielded from the solvent constitutes a permanent protection against aggregation. However, it is now clear that proteins can aggregate from their native states, being thus kinetically, but not thermodynamically, stable [23,24]. The present study suggests that, as for protein sequences, aggregation might influence the evolution of protein structures.

We acknowledge that the structures we studied here represent only a fraction of the soluble *E. coli* proteome. However, we expect that the conclusions we delineate would remain valid when more structures are available, since the significance of the observed differences was almost independent of the proportion of proteins selected to build up the subsets in the different analysis and, indeed, those relative to protein abundance are totally consistent with the ones obtained previously analysing a dataset of 397 *E. coli* structures, from which only 172 had abundance data [2]. It is important to note, however, that our analyses assume minor structural differences to exist between the used static structures and the conformation the respective proteins might adopt in the in vivo environment. Thus, it will be worth re-evaluating them when computational means would allow to model these functional conformations.

**Figure 13:**
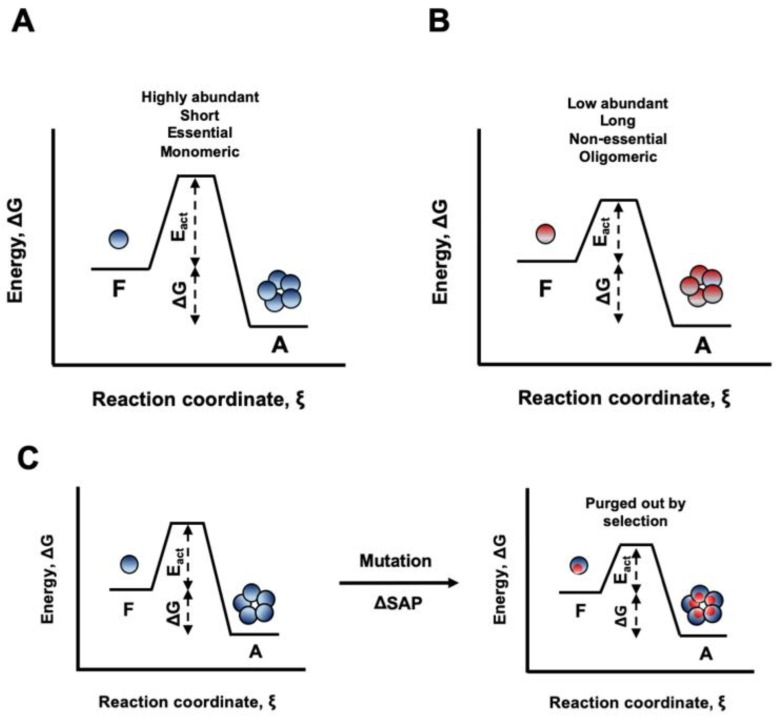
**Schematic representation of the energy diagrams for aggregation processes.** The ensemble of folded proteins in solution is considered as one thermodynamic system. The aggregated state represents in all cases the global minimum of Gibbs energy. However, to reach this state, proteins have to overcome kinetic barriers that differ in their activation energies (E_act_). (**A**) Proteins with low STAP, such as highly abundant, short, essential and proteins active as monomers are protected from aggregation by a high energy barrier. (**B**) Proteins with higher STAP, such as low abundant, long, non-essential and proteins active in oligomeric forms have to cross a lower energy barrier and thus, they can potentially access the aggregated state more frequently. (**C**) Mutations that increase the STAP without conferring additional functional advantages would be purged out by natural selection, since they decrease the energy barrier for aggregation from the initially folded state.

It has been suggested that, in the cell, globular proteins can remain soluble for long periods of time only because of the presence of high kinetic barriers that separate the native and the aggregated states [24]. Despite speculative, it is tempting to propose that the STAP of proteins has been shaped to keep these barriers high enough to allow them functioning at the concentrations required for an optimal cell fitness (Figure 13A,B). In this context, amino acid changes that, despite not affecting protein stability, would increase STAP, without providing a compensatory fitness benefit, would be purged out by natural selection, since they would decrease the energy barrier between folded and aggregated states and therefore potentially allow aggregation in a biologically relevant time (Figure 13C).

The study of the constraints imposed by protein aggregation on natural protein structures according to their abundance, function or tertiary/quaternary organization may uncover novel protein design principles with different potential applications. In this context, our analysis explains why the in vitro concentration of therapeutic globular proteins, like antibodies, above their natural abundance levels results, in many cases, in slow, but irreversible aggregation, precluding their marketing. Designed mutations that decrease the aggregation propensity of their protein surface, without impacting their stability, are anticipated to allow them to remain soluble and functional in the same conditions [65].

## Supplementary Materials

The following are available online at https://www.mdpi.com/2073-4409/8/8/856/s1, Table S1: List of low-aggregation and high-aggregation propensity operons, Table S2: List of the analysed structures together with the information about protein length, abundance, the presence of transmembrane segments, active form and essentiality.
