# Supplementary material for: Computational Assessment of Bacterial Protein Structures Indicates a Selection Against Aggregation
Source: Cells. 2019 Aug 8;8(8):856. doi: 10.3390/cells8080856 (PMC6721704; doi:10.3390/cells8080856)
Supplement: Supplementary file 1 [file cells-08-00856-s001.pdf]

Supplementary Material

Aggregation Propensities of Bacterial Protein Structures

Anita Carija[a+], Francisca Pinheiro[a+], Valentin Iglesias[a] and Salvador Ventura[a*]

Table S1. List of low-aggregation propensity operons (LA operons; blue), regulating proteins with structural aggregation propensity (STAP) lower than the mean aggregation propensity of the complete operon protein set (A3D average score = -0.880); together with the list of high-aggregation propensity operons (HA operons; red), regulating proteins with STAP higher than the mean aggregation propensity of the complete operon protein set. The average A3D scores of proteins regulated by the same operon are indicated, together with the number and % of proteins that could be identified as essential and non-essential.

LA operon names	A3D average score	nº proteins	EP	NP	Unknown
pheST-himA	-1.450	2	1	0	1
cmk-rpsA-himD	-1.329	2	0	0	2
prfB-lysS	-1.143	2	1	0	1
hslVU	-1.082	2	0	0	2
ptsHI-crr	-1.072	3	0	1	2
surA-pdxA-ksgA-apaGH	-1.055	2	0	1	1
def-fmt	-1.003	2	2	0	0
ribF-ileS-lspA-slpA-lytB	-0.993	2	1	1	0
fabHDG-acpP-fabF	-0.990	4	3	0	1
rpoE-rseABC	-0.965	2	1	1	0
rplJL-rpoBC	-0.955	2	2	0	0
moaABCDE	-0.936	2	0	1	0
iscRSUA	-0.930	2	0	0	2
metY-yhbC-nusA-infB-rbfA-truB-rpsO-pnp	-0.924	2	0	0	2
gcvTHP	-0.924	2	0	0	2
rbsDACBK	-0.919	2	0	2	0
glmUS	-0.904	2	2	0	0
hisGDCBHAFI	-0.900	3	0	0	3
panBC	-0.894	2	0	0	2
purMN	-0.893	2	0	1	1
pyrBI	-0.884	2	0	0	2
	**Total**	**46**	**13**	**8**	**24**
		**%**	**28**	**17**	**52**

HA operon names	A3D average score	nº proteins	EP	NP	Unknown
pdhR-aceEF-lpdA	-0.856	2	0	0	2
epd-pgk	-0.854	2	0	1	1
bioBFCD	-0.828	2	0	1	1
serC-aroA	-0.805	2	0	0	2
entCEBA-ybdB	-0.804	4	0	3	1
sdhCDAB-b0725-sucABCD	-0.791	4	1	2	1
accBC	-0.786	2	1	0	1
deoCABD	-0.770	3	0	1	2
mraZW-ftsLI-murEF-mraY-murD-ftsW-murGC-ddlB-ftsQAZ	-0.768	5	4	0	1
ydjA-selD-topB	-0.750	2	1	0	1
nrdAB	-0.748	2	2	0	0
araBAD	-0.744	2	0	0	2
nagBACD	-0.742	2	0	2	0
dhaKLM	-0.739	2	0	0	2
carAB	-0.722	2	0	1	1
galETKM	-0.693	2	0	2	0
rfaDFCL	-0.687	2	0	1	1
menFD-yfbB-menBCE	-0.656	2	0	0	2
malK-lamB-malM	-0.583	2	0	1	1
atpIBEFHAGDC	-0.375	4	0	0	0
cyoABCDE	0.034	2	0	2	0
	**Total**	**52**	**9**	**17**	**22**
		**%**	**17**	**33**	**42**

Table 2. The list of 619 analysed bacterial 3D structures indicated by their PDB ID together with the information about protein length, abundance, the presence of transmembrane segments, active form and essentiality. Aggregation propensity analyses were performed using sequential and structural predictors: AGGRESCAN and AGGRESCAN3D. Na4vSS values reflect the average protein aggregation propensities of the sequences and A3D average score indicate the structural aggregation propensity (STAP). For those proteins marked with *, the information regarding active form is not clear or is inexistent.

PDB ID	Protein length [aa]	Protein abundance [ppm]	Na4vSS	A3D average score	Presence of transmembrane segments	Active in monomeric form	Essential	Non-essential
1A04:A	216	457	-7,1	-0,8414		+		+
1A0P:A	298	1,85	-6,3	-0,8848		+		
1A16:A	441	357	-5,4	-0,8823				+
1A69:A	239	1345	2,7	-0,665				
1A6J:A	163	286	-7,9	-1,1471			+	
1A82:A	225	37,4	2,2	-0,6812				
1A99:A	370	453	-1,5	-0,9153				+
1ABE:A	329	262	-5,3	-0,9505		+	+	
1ACM:A	311	1398	-2,1	-0,7174				
1ACM:B	153	1664	-6,7	-1,0496				
1AF2:A	294	332	-2,8	-0,6252				+
1AF6:A	446	102	-10,9	-0,32	+			
1AG9:A	176	570	-9,3	-1,1207			+	
1AHH:A	255	456	-0,2	-0,741				+
1AI2:A	416	2246	-1,6	-0,8918				
1AJ0:A	282	24,1	-1,7	-0,8332				
1AKE:A	214	1371	-12,8	-1,0924		+		
1AMF:A	257	359	-1,4	-1,0172		+		+
1AON:P	97	3788	-2,1	-0,8172				
1AQ2:A	540	924	-5,9	-0,8076		+		+
1ARZ:A	273	230	-7,1	-0,9747				
1AZO:A	229	1,73	-3,1	-0,7509		+		
1AZY:A	440	129	-1,9	-0,7763				+
1B0A:A	288	89,8	1,6	-0,7797			+	
1B57:A	359	1877	-4,4	-0,7684				
1B6R:A	355	25,1	-0,9	-0,8315				+
1B6T:A	159	148	3,3	-0,7664			+	
1B93:A	152	203	3,7	-0,4936			+	
1B9L:A	120	149	-7,6	-1,2391				+
1B9M:A	262	36,7	-4,3	-0,9566				+
1BBU:A	505	600	-9,6	-1,0492				
1BCF:A	158	841	-11,8	-1,0395				
1BIQ:A	376	148	-1,1	-0,7109			+	
1BJN:A	362	1199	-0,4	-0,8276				
1BNC:A	449	222	-3,5	-0,7813			+	
1BO5:O	502	538	-4,2	-0,8071				
1BRM:A	367	852	0,4	-0,6725				
1BS4:A	169	241	-9,8	-1,2335		+	+	
1BSV:A	321	16,2	-3,5	-0,6732				
1BXR:A	1073	326	-4,2	-0,7109				
1BXR:B	382	557	-6,6	-0,7337				+
1C0N:A	406	75,2	2,4	-0,5942				
1C14:A	262	1336	3,4	-0,7801			+	
1C17:A*	79	324	41,5	1,05	+			
1C2T:A	212	64,4	-4	-0,9157		+		+
1C3B:A	377	4,5	1,9	-0,8229		+		+
1C5K:A	430	449	-4,5	-0,7535		+		+
1C7Y:A	203	56,2	1,5	-0,9978				
1C8U:A	286	82,7	-2,5	-0,695				
1CD5:A	266	212	-1,5	-0,8279				+

1CHN	129	397	-0,5	-0,908		+		
1CHU:A	540	6,84	-4,9	-0,7231		+		
1CKE:A	227	177	-5,4	-0,8669		+		
1CL0	321	341	-7,5	-0,9746				
1CL1:A	395	215	2,2	-0,6683				
1CLI:A	345	255	0,6	-0,87				
1CM5:A	760	3000	-6,8	-0,694				+
1CM7:A	363	452	-3,9	-0,9039				
1CMA:A	105	595	-21,3	-1,028				
1CS1:A	386	145	-1,4	-0,7441				
1D5N:A	206	4184	-9,5	-0,9886				
1D5Y:A	289	123	-2,7	-0,8674				+
1D8W:A	419	NA	-8,1	-0,7639				
1D9E:A	284	842	0,5	-0,7981			+	
1DC3:A	331	6295	-4,1	-0,7767			+	
1DCJ:A	81	48,6	-3,4	-0,9912		+		
1DFO:A	417	2620	-5,3	-0,7997				
1DKG:A	197	919	-17,5	-1,4018			+	
1DKL:A	432	5,57	-4,8	-0,7656		+	+	
1DM9:A	133	47,4	-27,3	-1,5561		+		+
1DO0:A	443	367	-12,8	-1,3555				
1DPE:A	535	1454	-7,3	-0,9083		+		+
1DTY:A	429	17,5	2,3	-0,6989				
1DU2:A	76	6,94	-11,6	-0,6673				
1DUD:A	151	207	-1,2	-0,5549			+	
1DUV:G	334	174	-6,1	-0,8245				
1E1O:A	505	203	-8,7	-0,8616				
1E3M:A	853	10,2	-6,4	-0,8455				
1E4O:A	797	119	-6,4	-0,8				+
1E58:A	250	2950	-11,1	-1,034				
1E5K:A	194	13,2	-4,5	-0,9095		+		
1E8C:A	495	94,5	-5,3	-0,8001		+	+	
1E94:A	176	115	-4,2	-0,8093				
1E9I:A	432	3360	-8,8	-0,8269			+	
1EBL:A	317	425	1,1	-0,699				
1ECB:A	505	97	-5,7	-0,792				+
1ECR:A	309	2,75	-9,7	-1,2606		+		
1EEJ:A	236	131	-1	-0,9698				+
1EGA:A	301	35,3	-1,6	-1,0541		+	+	
1EIX:A	245	48,1	-1,4	-0,8052				
1EK8	185	969	-22,6	-1,6259		+		
1EMD:A	312	NA	3,6	-0,773				
1EQ2:A	310	676	-1,3	-0,6283				
1EQR:A	590	509	-6,7	-1,0223				
1ESM:A	316	14,2	1,7	-0,7851			+	
1EUA:C	213	722	9,6	-0,8758				+
1EUG:A	229	25,9	-2,1	-0,7497		+		
1EUM:A	165	623	-8,8	-0,8685				+
1EW4:A	106	110	-19,4	-1,4974		+		
1EY1:A	139	164	-3,1	-1,1693		+		
1EYZ:A	392	184	0,6	-0,7603				
1F0K:A	355	31	0,4	-0,6659			+	
1F5V:A	240	109	-2,5	-0,7953				+
1F6D:A	376	24,7	-1,1	-0,9109				
1F9Z:A	135	213	-7,3	-1,0584				
1FC4:A	398	146	-4,3	-0,7901				+
1FC5:A	411	103	0,6	-0,7091				+
1FDY:A	297	45,1	3	-0,7267				
1FEP:A	746	168	-14,3	-0,2745		+		+
1FFT:A	663	62,7	36,9	0,26	+			+
1FFT:B	315	166	8,3	-0,19	+			+
1FG3:A	356	205	2,9	-0,7898				

1FIY:A	883	546	-5,4	-0,9018				
1FJJ:A	158	186	-4,9	-0,7123				
1FM0:D	81	39,3	1,5	-0,9213				+
1FMT:A	315	140	-1,2	-0,7726		+	+	
1FS0:E	139	625	-5,4	-0,8241				
1FUI:A	591	3,29	-6,8	-0,7251				
1FUO:A	467	465	-7,2	-0,7135				
1FW9:A	165	11	2,7	-0,7181		+		
1G0S:A	209	101	-5	-0,8157				+
1G6N:A	210	725	-1,5	-1,0579				
1G6S:A	427	148	0,1	-0,7833		+		
1G76:A	218	230	-11,8	-1,2344				+
1G8E:A	116	6,74	-4,1	-0,5733				
1G9S:A	178	220	4,1	-0,9441				
1GEU:A	450	110	-1,1	-0,735				
1GG1:A	350	533	-7,1	-0,7521				+
1GG4:A	452	39,6	0,7	-0,787		+	+	
1GGG:A	248	2340	-3,7	-1,196				+
1GIM	432	1502	-3,5	-0,8329				
1GLA:F	169	2174	3,2	-1,0175				
1GLG:A	332	2391	-12,7	-1,0197		+		
1GLV:A	316	150	-4,4	-0,9118				
1GMX:A	108	11,7	-8,4	-1,1253		+		
1GPM:A	525	616	1,1	-0,8626				
1GQE	365	287	-23,5	-1,2377		+	+	
1GQT:A	309	46	-4,9	-0,8509				+
1GRJ:A	158	583	-13,9	-1,2048		+		
1GRL:A	548	2932	-7,1	-0,9539			+	
1GTP:A	222	722	-1	-0,9297				
1GU6:A	478	17,7	-17,9	-1,0893				+
1GUB:A	311	31,4	-1,1	-0,9415		+		
1GVF:A	286	0,93	-0,2	-0,8531				+
1GVH	396	22,1	-6,5	-0,9066		+		
1GYT:A	503	185	-6,1	-0,8994				
1GYX:A	77	62,8	-9	-1,3351				
1GZ0:A	243	57,4	-2,6	-1,0387				
1H3D:A	299	285	-4,5	-1,0311				
1H5R:A	293	207	2,2	-0,8438				
1HJR:A	173	4,13	2,8	-0,7388				
1HO3:A	348	119	-4,1	-0,7852				
1HRU:A	190	60,2	-0,7	-0,9035				+
1HTT:A	424	360	-7,3	-0,998				
1HW1:A	239	134	-4,6	-1,1643				
1HXP:A	348	212	-11,8	-0,5893				+
1I01:A	244	951	-1,7	-1,0547			+	
1I1L:A	309	171	-0,2	-0,7319				+
1I52:A	236	8,14	-9,6	-0,8705			+	
1I6O:A	220	422	-1,8	-0,8543				
1I78:A	317	560	-10,2	-0,22	+			
1I7H:A	111	302	-17,4	-1,1964		+		
1I8D:A	213	194	-2,1	-0,7197			+	
1I8T:A	367	359	-3,5	-0,9219				
1IHF:A	99	2069	-26,4	-2,0298				
1IHF:B	94	1226	-22,7	-1,7919				
1IHO:A	283	463	-3,2	-1,097				
1IOV:A	306	37,7	3,7	-0,6792		+		
1ISA:A	193	705	1,3	-0,8923				
1IZY:A	283	539	-3,8	-0,8313				
1J79:A	348	372	-0,8	-0,8481				
1JDI:A	231	0,02	-4,3	-0,7811				
1JKE:A	145	89,2	-7,8	-1,1253				
1JKJ:A	289	1558	3,9	-0,6752				+

1JNS:A	93	303	0	-1,0651		+		
1JSW:A	478	691	0,2	-0,604				+
1JSX	207	24,5	1,6	-0,8672		+		
1JW2:A*	72	49,2	-7,6	-1,0843				+
1JXA:A	609	271	-2,8	-0,8801			+	
1JYH:A	157	111	1,1	-0,8943		+		
1JYS:A	232	152	5,7	-0,6994				
1K3F:A	253	1089	0,2	-0,6743				
1K75:A	434	213	-4	-0,8674				
1K7J:A	206	255	-4,5	-0,9163		+		+
1K7K:A	197	124	-7,3	-0,8819				
1KAG	173	185	-20,4	-1,245		+		
1KF6:A	602	188	-12,1	-0,7474				+
1KF6:C	131	39,3	35,8	0,81	+			
1KF6:D	119	1,38	43,4	0,94	+			
1KMI:Z	214	357	-18,4	-1,081				
1KMO:A	774	10,6	-11,9	-0,2964		+		+
1KON:A	246	389	-20,3	-1,3147		+		
1KP3	447	405	-8,1	-0,8255				
1KQA:A	203	5,63	2,1	-0,8037				
1KQF:A	1015	2,59	-11,7	-0,6521				+
1KQF:C	217	0,21	30,6	0	+			
1KS2:A	219	592	3,9	-0,7569				
1KSK:A	231	93,5	-10,9	-1,0477		+		+
1KTN:A	259	397	-4,7	-0,8672		+		
1KV7:A	516	75,3	-3,3	-0,5687		+		
1KY9:A	474	501	-5,2	-0,7909				+
1L5J:A	865	1511	-4	-0,7591		+		+
1L6W:A	220	82,8	8,7	-0,5597				
1L7V:A	326	NA	36,6	0,28	+			
1L7V:C	249	2,19	-6,9	-0,8596				
1L8A:A	887	1731	-8,9	-0,8148				
1LI5:A	461	218	-11,8	-0,8832		+		
1LOP:A	164	1040	-7,3	-1,0341		+	+	
1LQA:A	346	125	-5,9	-0,7753		+		
1M33:A	256	20,6	5,5	-0,7704		+		
1M3U:A	264	720	4	-0,6903				
1M5W:A	243	109	-9,9	-0,9535				
1M5Y:A	428	451	-14,3	-1,2188				+
1M65:A	245	37,8	-4,8	-0,8298			+	
1MBB:A	342	43,1	-3,3	-0,9347		+	+	
1MC3:A	293	12,8	1,7	-1,0824				
1MDQ:A	396	1391	-4,1	-1,0426		+		+
1MJC:A	70	717	-8,3	-1,0185		+		
1MKA:A	172	1064	3,4	-0,8614			+	
1MKZ:A	170	656	-8	-0,9516				
1MLA:A	309	782	1,1	-0,8055		+	+	
1MTL:A	168	105	-4,4	-1,0623		+		
1MUL:A	90	2594	-13	-1,1815				
1MXA:A	384	1081	-4,9	-0,7454			+	
1MZG:A	138	11,5	-3,1	-1,315				
1MZR:A	275	296	-8	-1,0478		+		
1N2Z:A	266	1,24	0,4	-0,9942				
1N3B:A	206	7,84	-6,3	-0,985		+	+	
1NAH:A	338	217	-6,5	-0,7966				+
1NAQ:A	112	40,5	0,6	-0,6013				
1NEK:D	115	222	62,8	1,31	+			
1NI5	432	11	-2,9	-0,8704			+	
1NI9:A	336	8,8	-6	-0,7571				
1NIJ:A	318	52,1	-0,6	-0,8808		+		
1NMN:A	138	15,6	-5,7	-1,153		+		
1NS5:A	155	26,4	-5,2	-0,8191				

1NUL:A	152	109	4,1	-0,5846				
1NXU:A	332	0,19	-5,3	-0,7781				
1NYE:A	143	1302	-7,2	-1,0063				+
1NYT:A*	272	43,6	2,7	-0,7721				
1O65:A	224	91,4	-6,4	-0,8194		+		+
1O89:A	324	344	-1,6	-0,7797				
1OG6:A	298	198	-0,4	-0,8291				+
1OI2:A	356	303	-5,2	-0,6103				
1OJ7:A	387	192	-3,4	-0,8454				+
1OK7:A	366	131	-2,8	-0,9504				
1OKJ:A	231	20,6	-2,1	-0,7956			+	
1OLT	457	10,5	-5,6	-0,8277		+		
1ONW:A	390	36,6	2,3	-0,5567				+
1OPD	85	1440	-7,2	-1,1786		+		
1OR7:A	191	56	-4,1	-1,1327			+	
1ORO:A	213	9,53	0,4	-1,0899				
1OY1:A	217	299	3,4	-0,6791				
1P3W:A	404	1189	-5,9	-0,8822				
1P9K:A	70	272	-2,1	-0,8049		+		
1P9P:A	255	15,4	-8,9	-1,0657			+	
1PFK:A	320	585	0,6	-0,8457				
1PHO:A	351	33,7	-13,3	-0,4767				
1PIL:A	112	274	0,4	-0,931				
1PKY:A	470	1279	-4,2	-0,7862				+
1PMM:A	466	523	-4,2	-0,7046				+
1POT:A	348	561	-6,3	-0,8933		+	+	
1PPY:A	126	117	-3,2	-0,8309				
1PS7:A	329	14,1	5,5	-0,761				
1PSD:A	410	891	-0,3	-0,8741				
1PSW:A	348	4,35	0,2	-0,7465		+		+
1PUG:A	109	1418	-21,6	-1,8508				+
1PUI:A	210	154	-12,6	-1,0095		+		
1PV4:A	419	2182	-7,2	-0,9289			+	
1PW4	452	45,5	29,5	0,04	+	+		
1Q16:C	225	1,24	33,7	0,51	+			
1Q2L:A	962	12,5	-6,7	-0,9478		+		
1Q5X:A	161	345	-1,5	-0,9327				
1Q6U:A	270	415	-17,2	-1,5349				
1QF6:A	642	797	-10	-1,0551			+	
1QFJ:A	233	77,9	-1,8	-1,0492		+		+
1QMH:A	338	0,03	3,1	-0,7049				
1QSA:A	645	63,5	-9,9	-0,9592		+		
1QXH:A	168	1831	6,1	-0,755				
1QYN:A	155	2261	-4,6	-0,7202			+	
1QYR:A	273	71,6	1,7	-0,8904		+		
1QYU:A	326	71,9	-10,5	-0,9111		+		
1R30:A	346	2,21	-11,3	-0,9739				+
1R3F:A	314	7,83	-9,2	-0,9306		+		
1R6Y:A	104	389	1,2	-0,7308				+
1R94:A	107	47,1	-5,1	-0,978				
1R9L:A	330	99	-6,4	-0,9664		+		+
1RBS	155	5,03	-17,3	-1,1792		+		
1RC2:A	231	NA	34,1	0,42	+			
1RC6:A	261	28,2	4,6	-0,488		+		
1RKQ:A	270	51,2	3,4	-0,6888				+
1RQI:A	299	46	-10,2	-0,8456			+	
1RWU:A	87	1011	3,7	-0,5628		+		+
1S1M:A	545	191	-0,7	-0,6553			+	
1S5T:A	292	265	0,3	-0,7532				
1S5U:A	134	36,3	7,4	-0,6644				+
1S96:A	207	91,6	-9,3	-0,9149				
1SB7:A	349	32,2	-7,9	-0,946		+		

1SDI:A	213	41,7	3	-0,763		+		
1SF2:A	426	242	-0,8	-0,7553				+
1SP5:A	242	50,5	-10,1	-0,9053		+		
1SQG:A	429	34,2	-9,7	-0,9894		+		
1SU1:A	184	139	1,9	-0,636				+
1T16:A	446	61,3	-4,8	-0,3495				
1T3D:A	273	80,5	0	-0,6206			+	
1T8R:A	484	47,1	-3,7	-0,7022				+
1TD2:A	287	41,8	5,2	-0,5811				+
1TE2:A	222	125	2,4	-0,8597				+
1TJ7:A	457	109	-8,8	-0,8652				
1TJL:A	151	408	-30,4	-1,7929		+		
1TLW:A	294	125	-3,4	-0,1586		+		+
1TQQ:A	493	219	-13,5	-0,93	+			+
1TRE:A	255	2956	-4,7	-0,7498				
1TXK:A	511	248	-8,7	-0,9226				
1TXL:A	216	237	-8,2	-1,3823		+		+
1TZP:A	274	5,67	-6,4	-0,7311				
1U08:A	386	8,47	1,6	-0,6595				
1UAE:A	419	102	0	-0,8571			+	
1UCW:A	317	1747	-5,6	-0,89				+
1UJ8:A	66	69,2	-18	-1,3899		+		+
1UQW:A	512	185	-2	-0,9304				
1URH:A	281	455	-2,9	-0,9667		+		
1URP:A	296	1391	-7,5	-0,9866		+		+
1USG:A	369	360	-8,5	-1,0315				+
1USH:A	550	122	-10	-0,887		+		+
1V57:A	248	27,1	0,5	-0,9309				
1V7Y:A	268	662	3,7	-0,8448				
1VB3:A	428	603	-2,1	-0,734		+		+
1VH1:A	248	194	-3,7	-1,0307			+	
1VHG:A	186	35,7	-1,8	-0,7744				+
1VIX:A	408	205	-5,1	-0,9057				
1VLY	326	372	-8,7	-0,8666		+		
1W26:A	432	1830	-14,4	-1,5984		+		
1W8G:A	234	171	-8,1	-0,8787		+		+
1WD6:A	101	529	-6,3	-1,2202			+	
1WNB:A	474	632	-0,6	-0,6783				+
1WXE:A	275	414	-9,4	-0,7568			+	
1X2A:A	396	1187	-5,6	-0,9004				+
1X2G:A	338	20,4	-8,7	-0,9106		+		
1XDP:A	688	51,5	-0,2	-0,851				
1XEY:A	466	1291	-3,6	-0,6932				
1XHS:A	113	74,6	-5,7	-1,0159		+		+
1XM5:A	155	87	-8,1	-1,043		+		
1XMV:A	353	443	-7,8	-1,1119				
1XQF	428	170	34,5	0,29	+			
1XS1:A	193	152	-4,6	-0,9626				
1XVI:A	271	138	-6,6	-0,7445				
1XVT:A	405	0,56	1	-0,723				
1XWY	260	5,58	-6,4	-0,8569		+		
1Y0G:A	191	91,7	-11,9	-1,2077				+
1Y79:1	681	190	-6,9	-0,7573		+		+
1YDY:A	358	211	-10,7	-1,0067				
1YIX:A	265	19,7	-6,7	-0,8357		+		+
1YJQ:A	303	3,5	-1,9	-0,8734		+		
1YRL:A	491	1659	-4,8	-0,9297				
1YT3:A	375	5,15	-0,4	-0,8855		+		
1Z15:A	367	2974	-7,3	-1,0527		+		
1Z9T:A*	243	49,9	-4,6	-0,699		+		+
1ZCD:A	388	6,86	39	0,19	+	+		
1ZFN:A	251	29,5	-5,9	-0,8266				+

1ZLQ:A	524	4,63	-2,4	-0,8558		+		
1ZMR	387	3692	-0,3	-0,93		+		
2ABH:A	346	429	-3,9	-1,0335		+		+
2AE0:X	365	8,05	-6,2	-0,855				+
2AJT:A	500	3,27	-3,6	-0,7059				
2AQ9:A	262	204	0,1	-0,7157			+	
2AU8:A	176	1634	-3,3	-0,8598				
2AVU:F	192	0,74	0,3	-0,711				
2B3D:A	193	177	-3,3	-1,0145				+
2B3T:A	360	32	-25	-0,927			+	
2BH7:A	276	4,78	-5,3	-0,8339				
2BON:A	299	35,3	-2,9	-0,8735		+		
2BTD:A	210	143	-4,2	-0,8668				
2BZ4:A	406	1127	-1,8	-0,6005			+	
2C44:A	471	1250	-0,2	-0,6351				
2C4N:A	250	86,2	6,9	-0,6565		+		+
2CXA:A	234	2,13	-0,2	-0,8481		+		
2D1P:A	128	29,8	1	-0,6692				
2D1P:B	119	17	7,8	-0,9409				
2D1P:C	95	16	4,2	-0,6687				
2D3W:A	248	105	-3,6	-0,8885				+
2DDM:A	283	70,8	5,4	-0,642				+
2DER:A	368	93,5	-6	-1,159		+		
2DHH:A	1049	48,1	13,4	-0,43	+			+
2DQ6:A	870	305	-6,4	-0,7439		+		+
2DXA:A	159	203	-3,4	-1,0545		+		+
2DY0:A	183	238	4,7	-0,8778				
2E3D:A	302	373	2,4	-0,7763			+	
2EA1:A	268	28,8	-11,2	-1,1272		+		
2EHJ:A	208	889	3,9	-0,963				
2EX2:A	477	4,11	1,1	-0,6914				
2EXW:A	473	1,53	24,7	-0,06	+			
2EYQ:A	1148	38,5	-7,1	-0,9942		+		+
2F00:A	491	55	-5,8	-0,832			+	
2F1C:X	301	0,52	-12,5	-0,27	+	+		
2F1F:A	163	332	0,1	-0,9879				+
2F9Y:A	319	669	-7,9	-0,7954			+	
2F9Y:B	304	299	-3,3	-0,6995			+	
2FCP	747	62,9	-9,8	-0,36	+	+		+
2FKB:A	180	164	-15,2	-0,9727		+		+
2FQ1:A	285	290	-5,5	-0,9105				+
2FRX:A	479	1,57	-3,6	-0,8421		+		
2FSH:A	901	255	-13,4	-1,0059		+	+	
2FWM:X	248	87,6	-3,7	-0,6206				+
2G8Y:A	361	199	-4,6	-0,7261				
2GFP:B	394	NA	34,1	0,39	+			
2GMW:A	191	32,4	-7,7	-1,0179		+		
2GQ1:A	332	190	-1,9	-0,7741				
2GQQ:A	164	1034	-7,6	-1,2024				
2GQR:A	237	771	-7,9	-0,9698				
2GUF	614	25,1	-8,2	-0,16	+	+		
2H5E:A	529	84,8	-7,1	-0,8873		+		
2HG2:A	479	3294	-4,3	-0,744				+
2HI7:B	176	28,3	33,7	1,23	+	+		
2HO9:A	167	285	11,7	-0,4207		+		
2HUR:A	143	2510	-3,2	-0,8222				
2HWG:A	575	1102	-6	-1,019				+
2I22:A	192	696	-4	-0,8737				
2ID0:A	644	128	-8,3	-1,0863		+		
2IGI:A	181	146	-8,7	-0,8456			+	
2IO7:A	619	72,2	-4,6	-0,7826				+
2IOP:A	624	421	-13,2	-1,2429				

2IS1:A	720	35,5	-11,9	-0,8863				+
2J0W:A	449	121	5,2	-0,7713				+
2J1N:A	367	678	-11,4	-0,37	+			
2J5V:A	367	114	-3,7	-0,817				
2JEE:A	81	2692	-35,6	-2,3732			+	
2JF4:A	565	263	-13	-0,8001		+		
2JGD:A	933	485	-9,9	-0,7472			+	
2JLC:A	556	6,8	-3,2	-0,6357				
2JO6:A	108	0,05	-3,1	-0,8677		+		
2JSX:A	87	0,7	-5	-1,2504		+		
2KDC:A	122	16,3	32,9	1,13	+			
2KFW:A	196	748	-21,9	-1,3321		+	+	
2KHO:A	638	4131	-18,2	-1,1646		+		+
2NYA:A	828	4,85	-8,5	-0,6941		+		
2O18:A	351	0,93	-6,1	-0,9804				
2O8V:A	244	139	-7,7	-1,0684				
2O97:B	90	4651	-11,8	-1,4295				+
2OAU:A	286	233	33,2	0	+			
2OGW:A	310	2599	-7,5	-0,8497		+		
2OI6:A	456	108	-7,4	-0,9285			+	
2OWO:A	671	42,3	-5,2	-0,9403		+	+	
2P2D:A	338	40	-1,8	-0,7388				
2P4B:A	318	89,6	-1	-0,7967				+
2P7V:A	158	258	-4,3	-1,2883				
2PAQ:A	199	12,2	-3,9	-0,8124				
2PJD:A	343	40,4	-2,4	-0,8183		+		+
2PTH	194	65,8	-3,9	-0,8629		+	+	
2PTS:A	456	460	-2,3	-0,8857			+	
2QCU:A	501	294	-5,7	-0,8011		+		+
2QFI:A	300	2,68	16,6	0,32	+			
2QRY:A	327	159	1,1	-0,9373		+		+
2QZS:A	477	236	4,9	-0,4924		+		
2R5N:A	663	644	-6,4	-0,7132			+	
2SCU:B	388	1380	-2,2	-0,8703				
2UVK:A	368	6,72	-6,7	-0,8596				
2UXT:B	470	3,23	-0,2	-0,5087		+		+
2UZZ:A	372	75,9	-5,4	-0,8969		+		
2V8N:A	417	7,7	45,7	0,35	+	+		
2V8Z:A	302	25,2	4,9	-0,6869				
2VEC:A	233	1,48	-7,6	-0,7568		+		
2VK2:A	318	324	-5,9	-0,9646		+		
2VYC:A	755	2,96	-4,5	-0,8124				
2W0Q:A	757	0,12	-6,9	-0,7668				
2WCD:A	303	4,77	-2,4	-0,94	+	+		
2WCI:A	115	860	-4,9	-1,0434			+	
2WCV:A	140	58,3	8,7	-0,6328				
2WDQ:A	588	487	-11,1	-0,737				
2WDQ:B	238	529	-5,1	-0,8711				+
2WDQ:C	129	76,3	37,9	0,92	+			
2WSX:A	504	0,03	40,8	0,05	+			
2WYL:A	354	24,4	-4,9	-0,7753				
2X26:A	319	4,49	-0,2	-0,8855		+		
2X5J:O	339	15,8	-1	-0,7785				+
2XHY:A	479	182	-1,6	-0,8122				
2XVA:A	197	178	-5,6	-1,1036				+
2Y4D:A	423	0,43	-9,9	-0,6997				
2YHS:A	497	215	-14,5	-1,1185		+	+	
2YXN:A	424	267	-7,7	-0,9946				
2ZPA:A	671	1,97	-6,6	-0,8184		+		
3A5Z:B	188	472	-4,5	-0,9967				
3A7L:A	129	161	-8	-1,0066		+		
3A8J:A	364	144	-3	-0,8409				

3ABO:A	453	13,3	-2,1	-0,5768				+
3AWI:A	668	4,57	-7,1	-0,8621		+		
3BF7:A*	254	98,2	-6,5	-0,9382				+
3BM1:A	183	284	-5,8	-0,8358			+	
3BT7:A	366	38,3	-5,8	-0,9955		+		
3C0K:A	396	29,6	-2,2	-0,7734				+
3CB4:A	599	196	-3,4	-0,9039		+		
3CDI:A	711	1266	-5,8	-0,9176				
3CES:A	629	27,2	-8,9	-0,7474				
3CUZ:A	533	1625	-6,4	-0,7936		+		
3DHW:A	217	19,1	44,3	0,64	+	+		
3DHW:C	343	89,6	-1,4	-0,9681				
3DJL:A	541	22,8	-2,4	-0,627				
3EFP:A	204	8,5	3,8	-0,6142		+		
3EH0:A	341	177	3	-0,77			+	
3EJE:A	78	2944	-18	-1,4019			+	
3F4L:A	345	87,4	-4,2	-0,8448				+
3F7T:A	316	61,9	-6,5	-1,0378			+	
3FFV:A	181	18,3	-4	-0,9847		+		
3FMT:A	181	156	-5,3	-1,1826			+	
3FPP:A	371	13,7	-6,1	-1,23	+			
3H94:A	407	60,3	-0,7	-0,9146		+		
3HW9:A	362	694	-7,3	-0,41	+			+
3HWO:A	391	132	-6,9	-0,871		+		+
3IPO:A	435	28,9	-10,8	-0,8404		+		+
3IYD:A	329	1140	-7,7	-0,9855			+	
3JSP:A	202	51,8	-9,4	-1,1916			+	
3K07	1047	3,98	15,3	-0,42	+			
3L9W:A	176	15	0,4	-0,7914				
3LBF:A	208	20,9	-4	-1,0079		+		
3M9W:A	330	169	-5,2	-1,0651		+		
3MMP:A	283	3012	-7,5	-1,0644			+	
3MT6:E	207	732	-1,6	-0,7372				
3N1S:A	119	679	-0,1	-0,8167				+
3N75:A	715	8,98	1,4	-0,7724				
3NBU:A	549	800	-2,9	-0,8282				
3NBX:X	498	16,4	-3,9	-0,9753				
3NKD:A	305	2,75	3,6	-0,9124				
3NZQ:A	658	199	-5,9	-0,902				
3O4F:A	288	96	-0,8	-0,5139				
3O7Q	438	0,08	32,5	0,15	+	+		
3O9P:A	537	109	-3,4	-0,8383		+		+
3OAA:A	513	1622	-1,7	-0,789				
3OAA:G	287	846	-4,5	-0,9404				
3PCO:B	795	260	-3,4	-0,969			+	
3PIK:A	457	65,2	-6,4	-0,7494				
3PRW:A	392	199	0,8	-0,5233		+		+
3Q9L:A*	270	657	-4,1	-1,0306			+	
3QE7:A	429	24,2	40,6	0,33	+	+		
3QOU:A	284	394	-9,8	-1,1626		+		+
3QXF:A	368	0,27	-8,1	-0,8644		+		
3R1R:A	761	390	-4,7	-0,7848			+	
3R2Q:A	202	125	2,8	-0,8311				+
3RFA:A	384	46,1	-7,4	-1,008		+		+
3RFZ:B	878	0,99	-8,5	-0,5646				
3RLF:A	371	158	1,1	-0,8448				+
3RLF:F	514	1,33	20,8	-0,06	+			
3RLF:G	296	0,06	36,5	0,44	+			
3SH0:A	253	38,8	-12,5	-1,1777				
3SS7:X	442	14,7	-1	-0,7675		+		
3T36:A	203	4,88	-3	-1,0608		+		
3T89:A	285	219	-9,7	-0,6753				

3TAT:A	397	211	2,2	-0,8154			+	
3TCF:A	543	1186	-6	-0,8205		+		+
3TG0:B	471	19,6	-15	-0,7955				
3TGO:A	245	151	-9,2	-0,9334		+	+	
3TGO:C	344	269	-10,2	-0,6367		+		
3TKA:A	313	84,4	-12,6	-1,1138				
3TLK:A	318	53,5	-4,3	-0,9371		+		
3TPV:B	440	4,31	0,6	-0,7421				+
3U0O:A	347	191	0,4	-0,6639		+		
3UAG	438	59,4	-2,6	-0,7787		+	+	
3V8V:A	702	19,9	-8,3	-0,9047		+		+
3VH0:A	353	353	-8,3	-0,9203		+		
3VU3:A	753	529	-13,3	-0,7251				
3WLX:A	333	130	-6	-0,7664				
3WRP:A	108	220	-12,5	-1,3771				
3ZGZ:A	860	430	-6,2	-0,9539		+		
4A2C:A	346	481	6	-0,6166			+	
4AQ4:A	438	474	-10,5	-0,8672				
4ATN:A	366	14,8	-1	-0,8846		+		
4BHT:A	447	663	-4,3	-0,7765				+
4BIN:A	417	1,29	-5,3	-0,764		+		
4C00:A	577	9,82	-4,5	-0,57	+	+		
4C5F:A	359	5,77	-3,2	-0,84		+		
4CSU:9	390	215	-15	-0,873		+	+	
4CVQ:A	405	74,5	1	-0,7816				
4D02:A	479	0,24	-7,2	-0,8195				
4D8J:A	150	126	-18,2	-1,5502				
4DCM:A	378	1,29	-2,9	-0,7242		+		
4DJK:A	511	173	38	0,19	+	+		
4DT4:A	149	255	-6,8	-0,9488		+		+
4E8B:A	243	10,5	-7,1	-1,052				
4F9J:A	672	0,04	-3,1	-0,8314		+		
4FIS:A	98	224	-12,9	-1,1477				
4G6B:A	427	768	-1,6	-0,8721				+
4GC0:A	491	0,06	30,8	0,08	+	+		
4GO1:A	317	11,2	1,7	-0,8702				+
4HG0:A	292	116	-12,6	-1,2267				+
4HR7:A	156	218	-1,4	-0,7893				
4IJZ:A	274	69	-2,3	-0,8248		+		
4IR1:A	351	NA	-4,9	-0,9707		+		
4IU9:B	462	3,2	35,3	0,17	+	+		
4IWN:A	247	41,1	-1,7	-0,9053		+		+
4IZ6:A	536	113	-2,6	-0,8132				
4JAK:A	157	6,82	-1,4	-0,7902				
4JDR:A	474	1495	-0,4	-0,8969				
4JHC:A	194	108	-9	-1,0064				
4JK1:C	1342	967	-9,3	-0,9893			+	
4JR9:A	463	2,43	30,7	0,05	+			
4JXD:A	113	0,04	4,5	-0,914		+		
4K4C:A	137	77	-9	-0,9711				
4KDC:A	240	73,8	-3,1	-0,7192				
4KMU:D	1407	690	-6,1	-0,9197			+	
4KN7:E	91	1644	-23,4	-1,6443				+
4KNZ:A	264	147	-4,9	-0,947				
4KX4:A	215	465	-4,5	-1,05		+		+
4LGT:A	291	76,1	-16,3	-1,1718				
4MZ9:A	178	404	-25,7	-1,0982			+	
4O5Q:A	521	268	-4	-1,009				
4OBY:A	577	152	-6,1	-1,0282		+		
4P0E:A	197	11,8	-8,1	-0,8733				
4P1M:A	109	145	-26,2	-1,6892				
4P31:A	241	187	-4,4	-0,9997		+	+	

4Q65:A	493	0,01	33,5	0,23	+	+		
4Q86:A	586	15,5	-2,6	-0,7948				+
4QR8:A	443	322	-1,5	-0,6945				+
4R9M:A	186	45,3	-3,7	-1,0062				+
4RP9:A	465	NA	40,2	0,11	+			
4S20:K	968	46,3	-10,7	-0,9896				
4TKY:A	208	314	2,5	-0,9988		+		
4TMK:A	213	42,9	-5,5	-0,9609			+	
4V2S:A	102	1333	-14,2	-0,732			+	
4WQ4:A	337	10,9	-1,7	-0,6753			+	
4YG2:F	613	306	-19,2	-1,4577			+	
